# Supplementary figures and images for: Demarcating geographic regions using community detection in commuting networks with significant self-loops
Source: PLoS One. 2020 Apr 29;15(4):e0230941. doi: 10.1371/journal.pone.0230941 (PMC7190107; doi:10.1371/journal.pone.0230941)

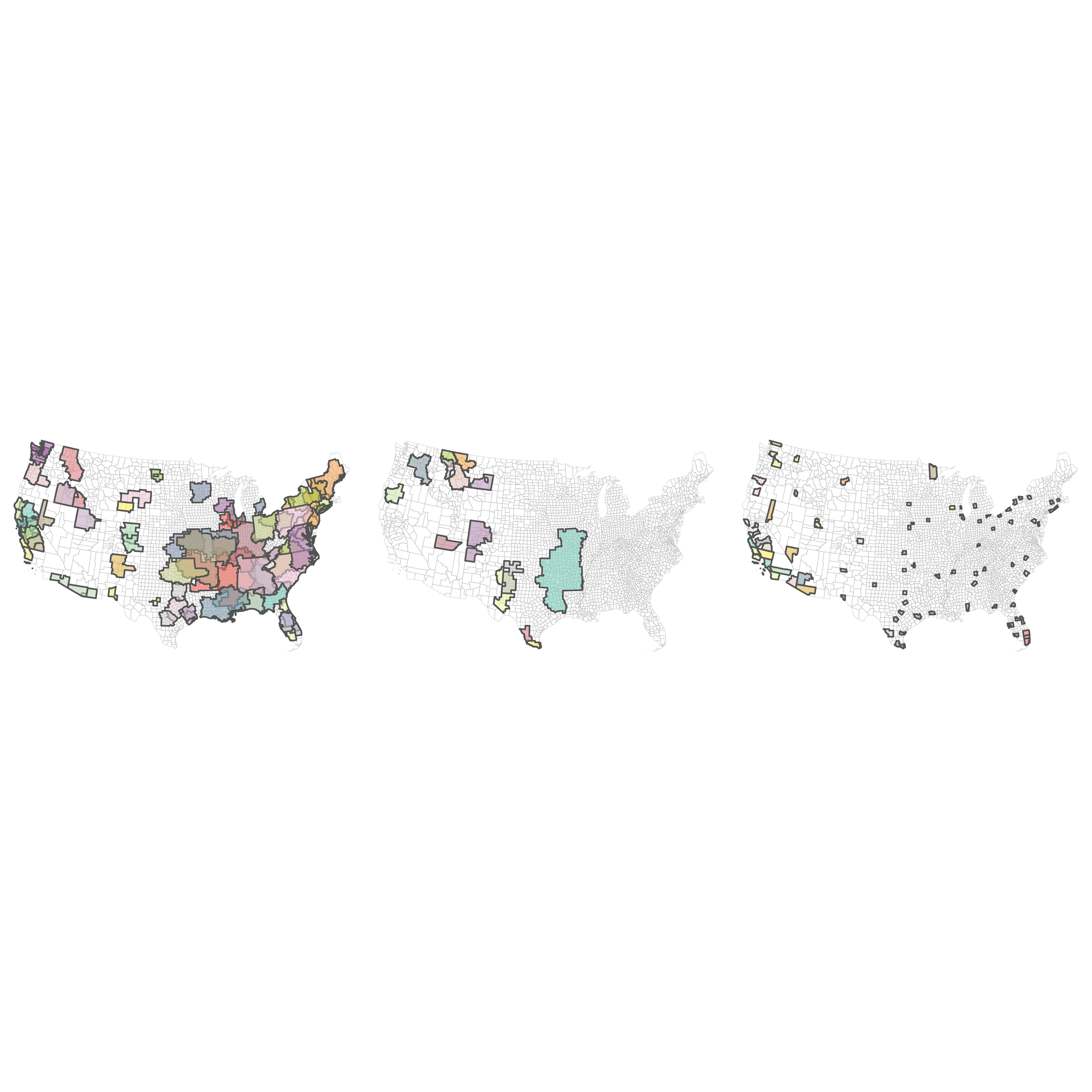

Supplement: S1 Fig — Communities (non-nodal) (left), nodal clusters (middle), and monads (right). (TIFF) [file pone.0230941.s001.tiff]

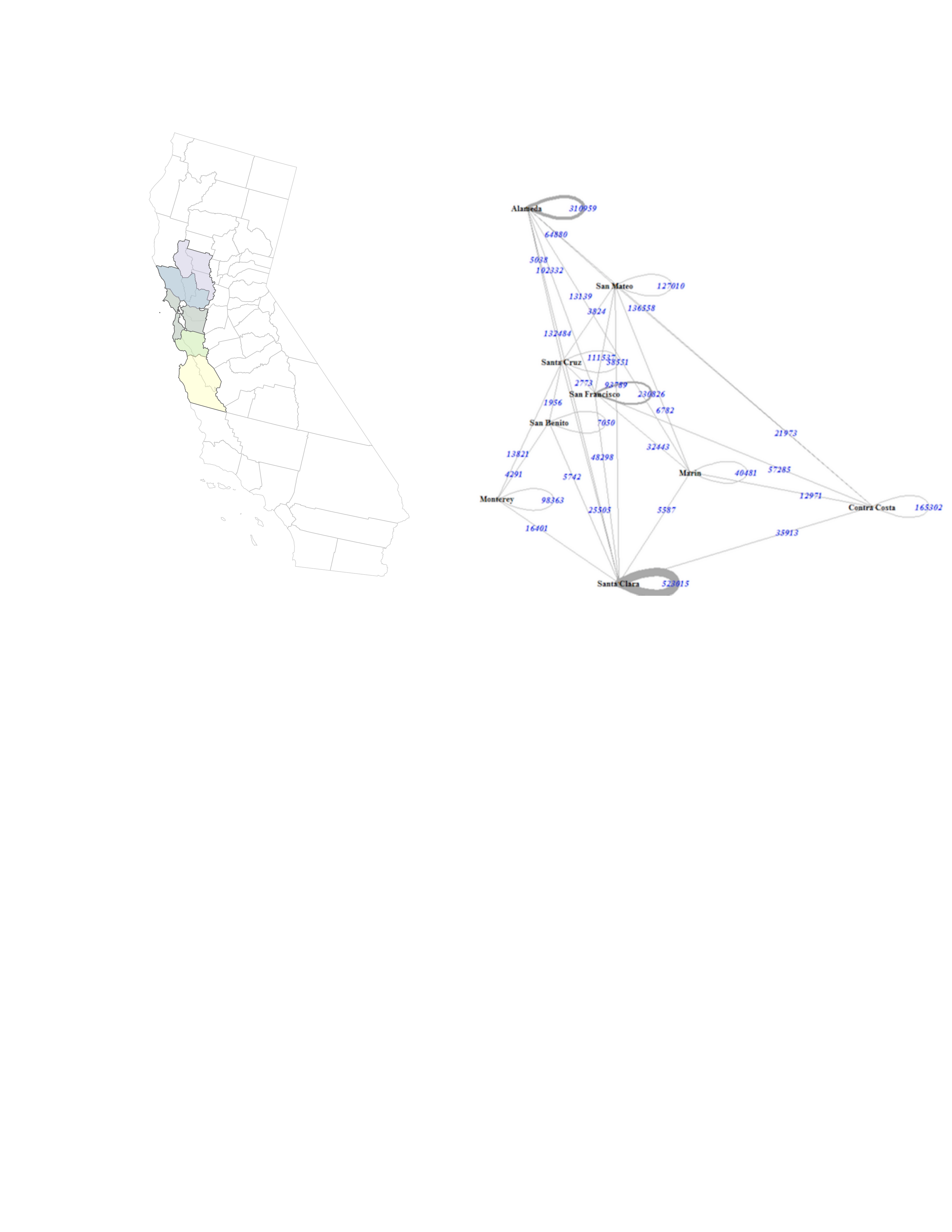

Supplement: S2 Fig — (TIFF) [file pone.0230941.s002.tiff]

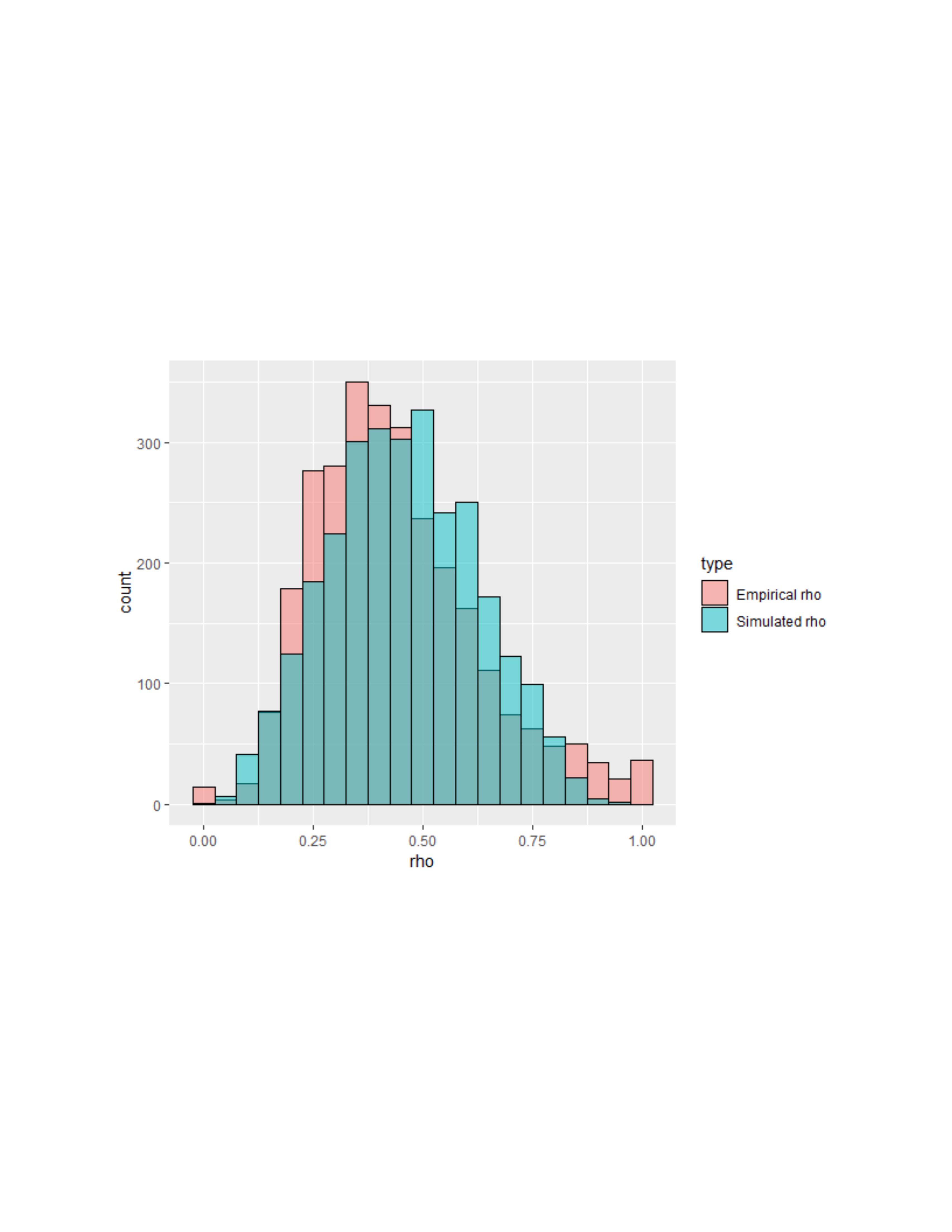

Supplement: S1 File — (ZIP) [file pone.0230941.s003.zip › si/B1_rho_histogram.tiff]

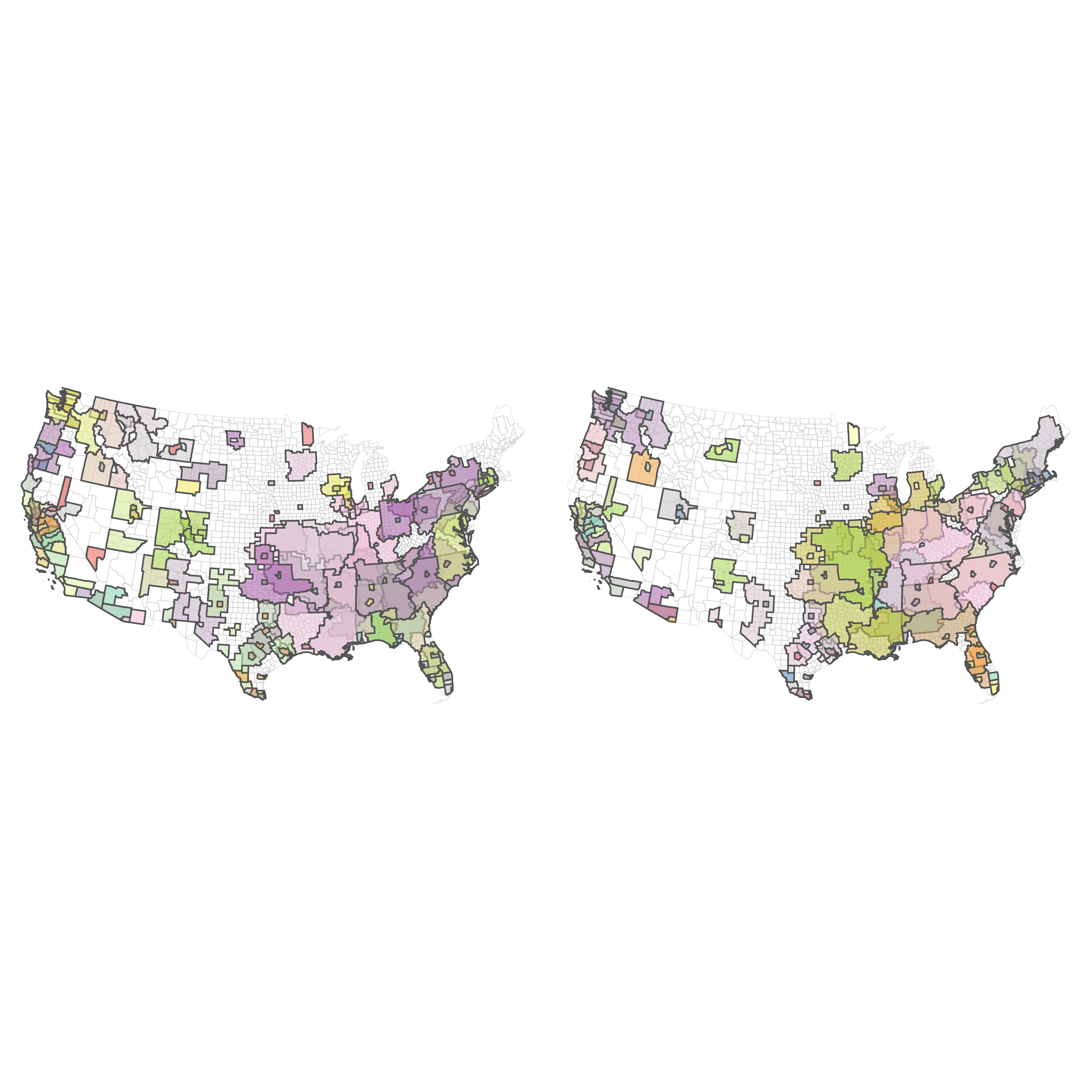

Supplement: S1 File — (ZIP) [file pone.0230941.s003.zip › si/B4_diffinit.tiff]

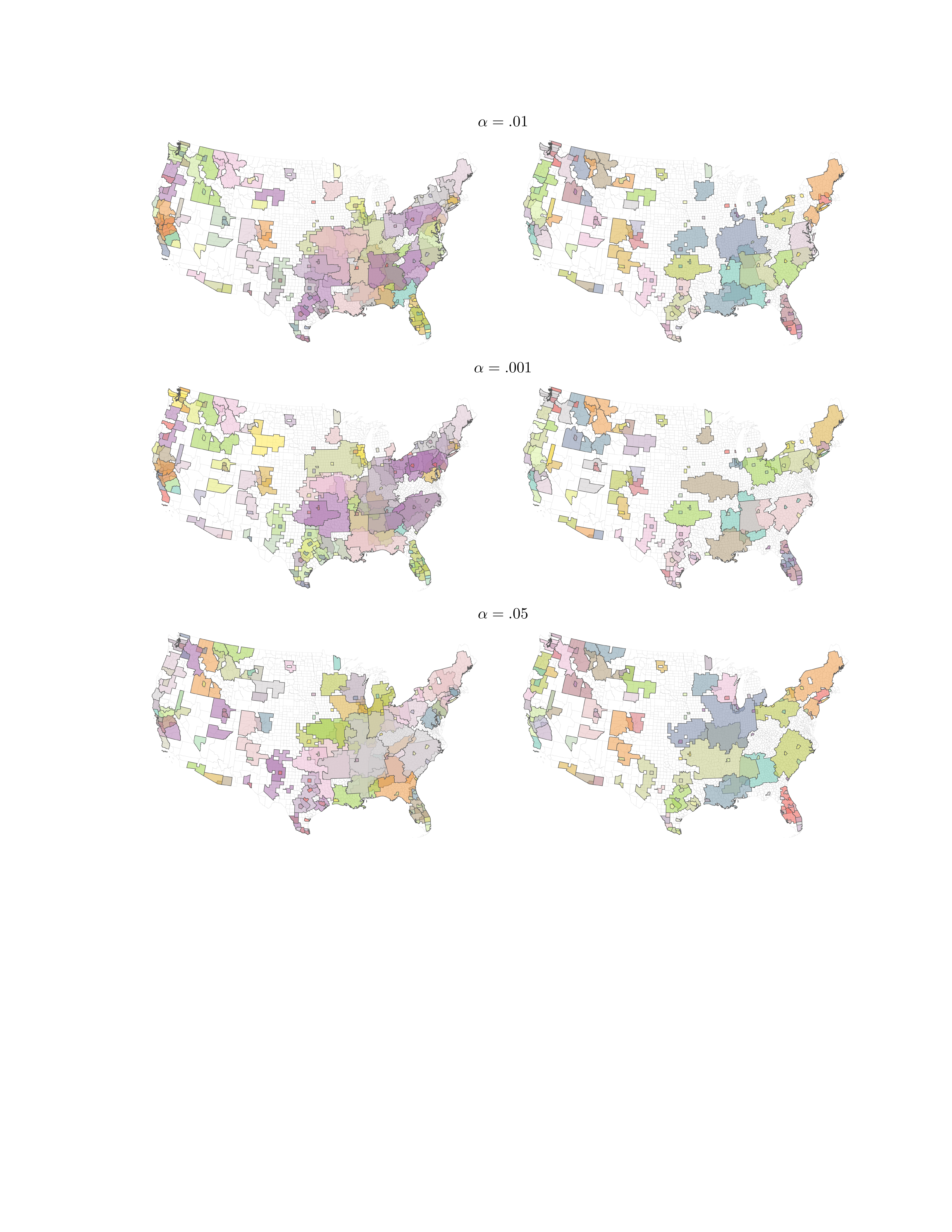

Supplement: S1 File — (ZIP) [file pone.0230941.s003.zip › si/B3_compare_Sensitivity.tiff]

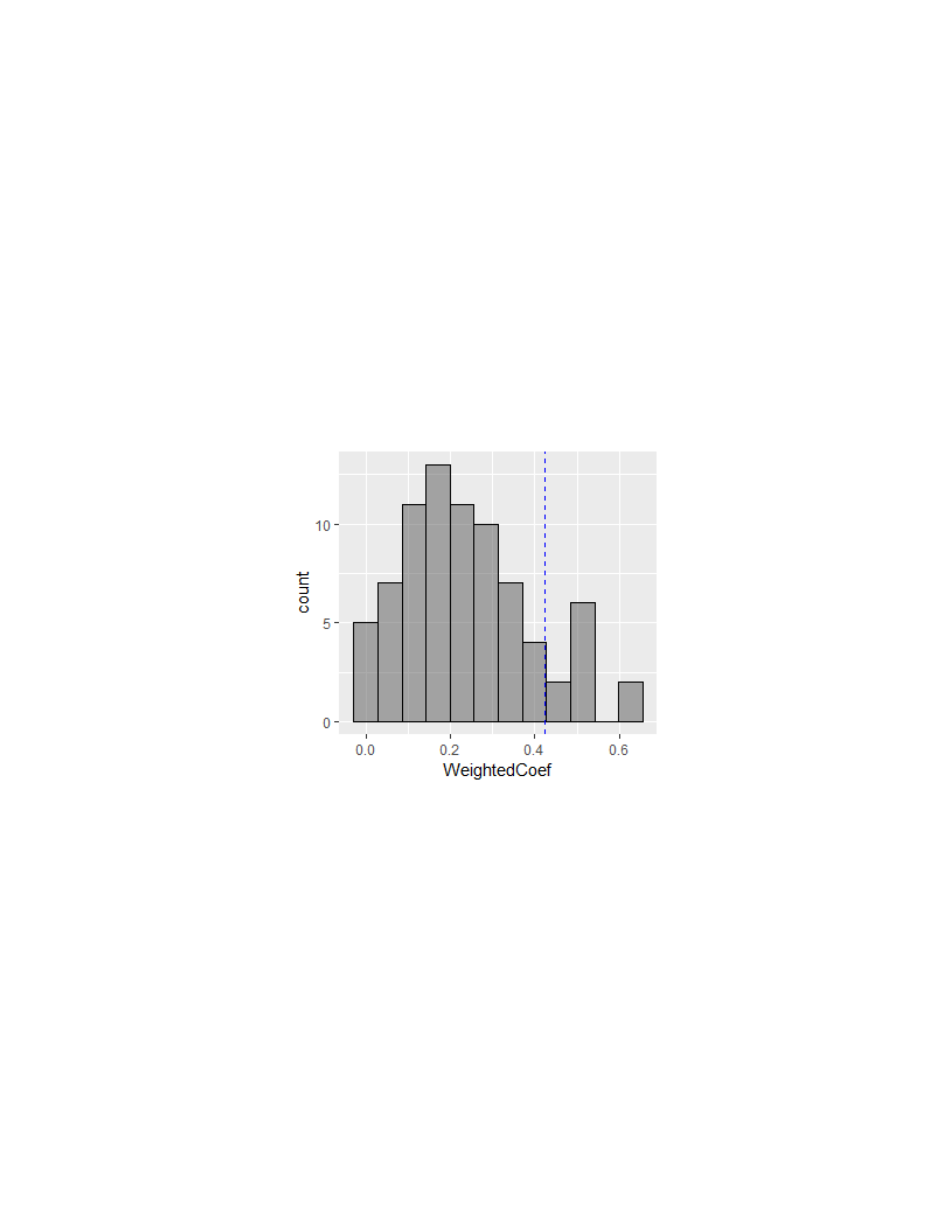

Supplement: S1 File — (ZIP) [file pone.0230941.s003.zip › si/B2_mono_hist.tiff]
