# Supplementary material for: Demarcating geographic regions using community detection in commuting networks with significant self-loops
Source: PLoS One. 2020 Apr 29;15(4):e0230941. doi: 10.1371/journal.pone.0230941 (PMC7190107; doi:10.1371/journal.pone.0230941)
Supplement: S1 File — (ZIP) [file pone.0230941.s003.zip › si/si.pdf]

1 Supplementary Figures

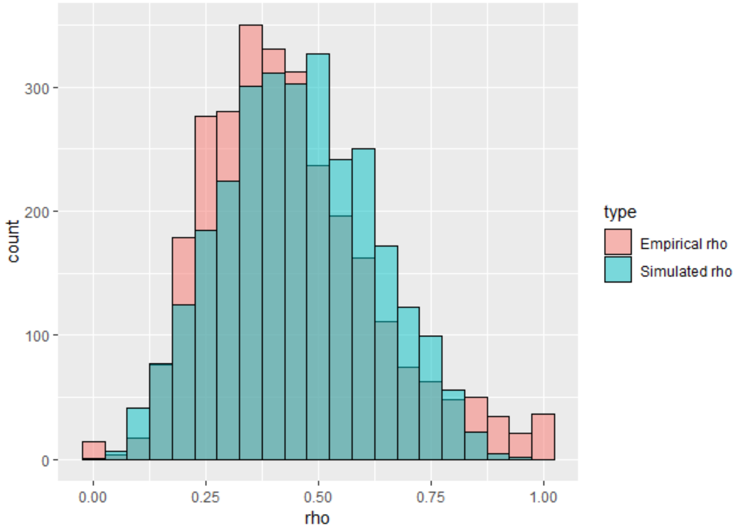

Figure 1: Histogram of comparison of ϱ_u with simulated data generated from Beta$(\hat{a}, \hat{b})$. In practice, $\hat{a}$ and $\hat{b}$ are respectively 3.86 and 4.50 for the commuting dataset in this study.

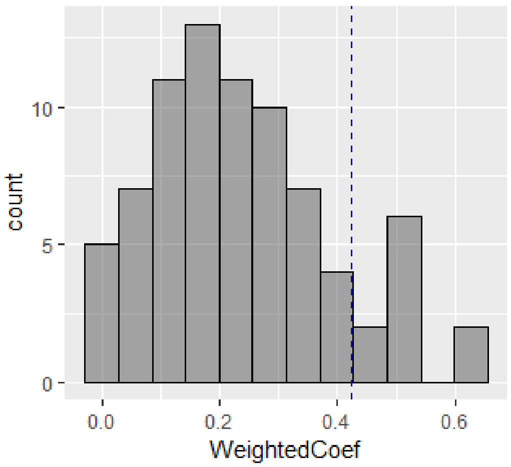

Figure 2: Histogram of weight clustering coefficients as described in section 4.5.

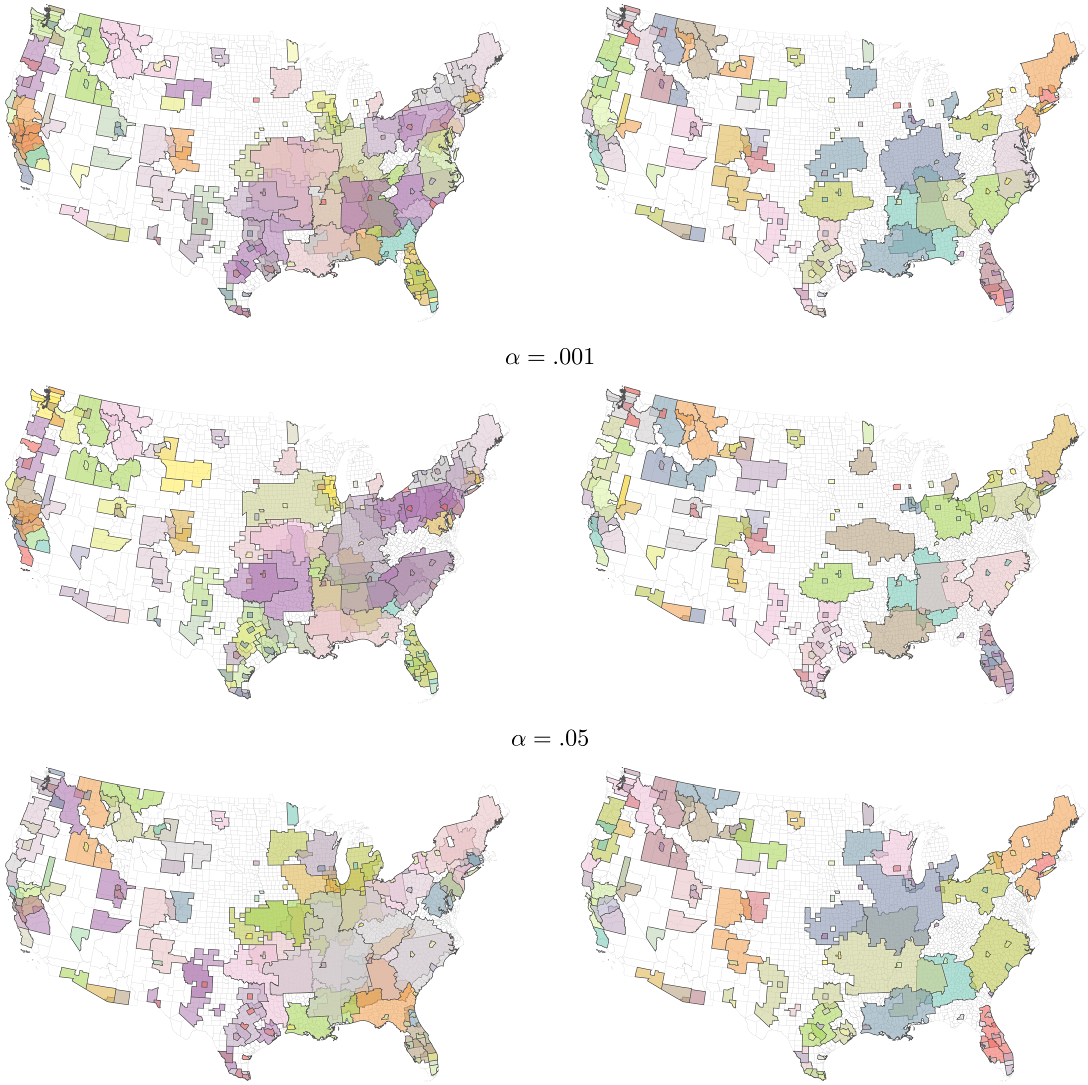

Figure 3: CCME-SL algorithm run under varying constraints for τ (*left*: $\tau = .8$,*right*: $\tau = .5$) and α. Decreasing the overlap parameter τ and the significance parameter α in general yields lower coverage of the total counties.

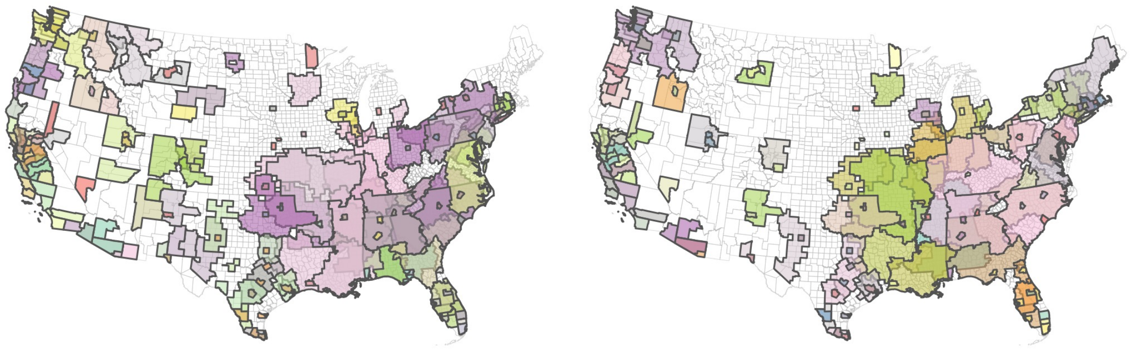

Figure 4: CCME-SL algorithm run under differing initializations using thresholding of self-loops (*left*: $W_{uu} > 10,000$,*right*: $W_{uu} > 50,000$ and α. The resultant communities do not differ much from those initialized with the threshold 20,000
