# Supplementary material for: Demarcating geographic regions using community detection in commuting networks with significant self-loops
Source: PLoS One. 2020 Apr 29;15(4):e0230941. doi: 10.1371/journal.pone.0230941 (PMC7190107; doi:10.1371/journal.pone.0230941)
Supplement: Attachment — Submitted filename: RESPONSE_2.pdf [file pone.0230941.s004.pdf]

Response to Reviewer 1

1 Introduction

We thank the referee for reading through the paper. We also like to thank you for pointing out the various issues in the original manuscript. Your detailed comments have furthered our own knowledge of the field and given us a better perspective of both the position of the methodology developed in this paper in context of the general area of community detection as well as increased our knowledge of related work on community detection and its applications in the general field of spatial network analysis. We have gone back and read through the paper closely and tried to correct as many issues as we could find.

Brief comment on the revision:

We have gone through and revised the entire paper. We have tried to make the revision and in particular the changes made in the revision as easy as possible to spot. All changes made in the manuscript can be found in this color.

If the colors are distracting please let us know and we are happy to upload a version without these colors.

2 Changes made in light of the report

Here we address changes made in the revision in light of the report. Below, text presented in grey quotes a given review verbatim or editorial comment, in its entirety, and in the order of the review. Following each quote, we address the comment by discussing how we improved our manuscript.

2.1 Changes following Minor comments

1. Q1: I think that the method is technically sound from the point of view of the mathematics in it, but I do not think the data support the conclusions. I disagree that including self-edges is novel in the field of community detection (although towards the end in Section 6.4 the authors explain that they mean the use of community detection to study commuting networks). I also believe that one of the biggest points that the work argues is that the proposed method has a high coverage of the commuter travels, but I fail to see why that is the determinant justification of the method working. For example, the coverage can be trivially maximised by taking a single community that covers all of the US, but such a community is obviously not relevant. This fits into the larger issue of validation, of how can the communities found be interpreted (a general problem in the field of community detection) and I think that finding statistically significant will only give you confidence with respect to the thing you are trying to explain (coverage as compared to the null model). I am sceptical that some of the largest communities (such as the light blue in fig 2b) are useful. Finally, I think that the claims made in the conclusion about dramatically changing our understanding of the metropolitan structure of the US are not supported by the work presented before.

   Addressed:

   We have added several figures and sections in the body of the text arguing the validity anmd reliability of the *CCME-SL* method. Notably, we compared the performance of the method to degree-corrected stochastic blockmodel and modularity maximization (figure 6) and discussed the comparison of the results in section 6.2 (paragraph 456). We also analyzed the sensitivity of the method over a range of parameters and initializations in figures B.9 and B.10 in the supplement and discussed these results in sections 7.4 and 8 (approximately paragraph 505 and 518).

Coverage rate is but one way that we have compared the resultant clusters from the proposed method to existing MSAs and megaregions. However, the primary purpose of this article is not to argue that our proposed method is adfvantageous in any single objective metric, as such a metric would not exist (as suggested by the reviewer) , but to explore the ways that the proposed method may glean more information.

We have changed some of the wording in the body of the text to mute the potentially hyperbolic claims, as pointed out by the reviewer. However, we do asert that the regions found by the proposed method offers an alternattive and potentially more useful way than traditional delineations.

We have expanded discussion and motivation for exactly why larger regions found by CCME-SL in the inrtoduction and related work sections (sections 1 and 2, paragraphs 67- 69). CCME-SL finds larger regions than existing delineations is useful because regional policy is often enacted and implemented depending on Metropolitan delineations, which have real impacts for govermental programs as well as a research that use metropolitan boundaries as given.

2. Q2: I think that the statistical analysis was done rigorously. However, something that is not entirely clear to me from the text is if the CCME algorithm is proposed here or if it has been proposed somewhere else (in which case a citation would be needed). It's not entirely clear why the calculation of the variances is relevant for the analysis and it should be explained in the text (and I am not recommending to remove the calculations). In section 7 the text says that the sensitivity analysis can be found in the supplementary material and this is not the case.

   Addressed: Excellent point.

   Many thanks for this comment. In light of the comment we have

   (i) Given a brief review of these type of null models. A similar null model was developed by one of the authors (Bhamidi) in an earlier work but in a simpler setting and the differences are now described in Section 4.2.1, which gives background on the previous model *CCME* and 4.2.2, which describes the newly proposed model *CCME-SL*. (paragraphs 170-209)

   (ii) We have now removed the explicit calculations of the variance and these can be found in Appendix A. However we have now given explanations for the need for these parameter estimates in that Section.

   (iii) We have included in the sensitivity analysis in the appendices section, which vary the input parameters α and τ. We have also fixed the text in section 7 (approximately paragraph 518) to reflect this.

3. Q3: As far as the material that was available to download as an editor, I did not have access to any type of data. This includes data of commuters, networks build, maps, figures and anything that would be needed to reproduce the analysis.

   Addressed: We thank the reviewer for addressing the concerns. We have uploaded our data, code, and supplemetary materials on Github in the repository titled 'Commuting Community' under the user *markhe1111*. The url for all of these materials is `https://github.com/markhe1111/commuting-communities`

4. Q4: The article is written in generally good English but it has not been revised with care. There are many instances of missing words or extra words, specially towards the end of the article. More citations should be added, for example for the **Benjami-Hochberg procedure**. One further comment is that the terminology that is new to the article should be presented at the beginning, not in Section 5 (Results) as is the case for non-nodal community, monad, etc. While I agree that it is necessary to distinguish the different types of communties found, I think that in the context of the field is is confusing to use the work cluster to mean a community that contains more than one node. To the best of my knowledge, the word monad has been introduced in this manuscript and this is also a non-standard term. I would prefer the **self-explanatory single-node community.**

Addressed: Many thanks for this comment, this has lead to a significant improvement in the exposition of the paper. In light of the above comment we have

(i) Reread the entire paper and improved the presentation.

(ii) Included reference on controlling false discovery and a paragraph explaining this in Section 5 when describing the algorithm.

(iii) We have now given an explicit definition of the types of communities that the method derives in the very second page in the introduction of the method.

Finally regarding the phrase “Monad” we are not strongly tied to this phrase but wanted to evoke the notion of a vertex that still has a strong propensity for self-loop even after accounting for baseline self-loop. It is also chosen on the basis of its analogous yet contrasting relationship to ‘dyad’ which describes a exclusively strong tie between two nodes. While we are not wedded to this term, we think self-explanatory single-node community is a mouthful.

2.2 Other changes

We now describe the other changes in the revision: we have included a survey of related literature on community detection as applied to spatial and urban network data (section 2.2, starting from paragraph 105). We have added a review of the literature at the end of section 4.2 (paragraph 168 (approximately)) that discuss the existing literature on self-loops. We have given an overview of the relationship between the method in this paper and it’s place in the general context of community detection methodology (7.4, paragraph 509). We have addressed the *resolution limit* in this section as well as in the *limitations and further research* sections. (section 8.1, paragraph 520).

We validated the method thoroughly by comparing its performance to other methods. We added figure 6 and section 6.2 (paragraph 459), which compares CCME-SL with degree corrected stochastic blockmodel, the modularity algorithm (Louvain), as well as existing delineations. We have moved the map showing delineations form MSAs and megaregions to figure 6 from figure 2. We have also added communities under different initialization conditions alongside the communities clustered under various parameters (which we have moved to the supplemental figures in point 2) in the supplement.

Response to Reviewer 2

Introduction

We thank the referee for reading through the paper and for the enthusiastic reception. We also like to thank you for pointing out the various issues in the original manuscript. Your detailed comments have furthered our own knowledge of the field and given us a better perspective of both the position of the methodology developed in this paper in context of the general area of community detection as well as increased our knowledge of related work on community detection and its applications in the general field of spatial network analysis. We have gone back and read through the paper closely and tried to correct as many issues as we could find.

Brief comment on the revision:

We have gone through and revised the entire paper. We have tried to make the revision and in particular the changes made in the revision as easy as possible to spot. All changes made in the manuscript can be found in this color.

If the colors are distracting please let us know and we are happy to upload a version without these colors.

Changes made in light of the report

Here we address changes made in the revision in light of the report. Below, text presented in grey quotes a given review verbatim or editorial comment, in its entirety, and in the order of the review. Following each quote, we address the comment by discussing how we improved our manuscript.

(1) The authors identified the clusters of counties in US from commuting data among them, using by a overlapping community detection method with self-loops. Such a data-driven approach to identify spatial units would be promising, overcoming the problems of the traditional heuristic methods. On the other hand, this is not the first paper for this approach, and the reliability of their method should be checked carefully to use the results for statistical, governance and planning purposes that the authors argued in the paper.

Addressed:

We have conducted several comparative analyzes to thoroughly address the issue of reliability. We added a new figure (figure 6) in section 6.2 (starting approximately at paragraph 456) which compares the results from the proposed method to degree-corrected stochastic blockmodel as well as the modularity algorithm. We have added figures showing the performance of the algorithm under several different tuning parameters as well as differing initialization conditions (figures B.9 and B.10 in the supplement) with descriptions in sections 7.4 and 8 (approximately paragraph 505 and 518) produces comparable results across different parameters to verify that the method is reliable.

(2) There are some papers to identify spatial units from the human mobility datasets. For examples,

(a) - The impact of social segregation on human mobility in developing and industrialized regions by Amini, A., Kung, K., Kang, C. et al. EPJ Data Sci. (2014) 3: 6. https://doi.org/10.1140/epjds31.

(b) - Comparing Community Detection Algorithms in Transport Networks via Points of Interest by Huang, Liping & Yang, Yongjian & Gao, Hepeng & Zhao, Xuehua & Du, Zhanwei. (2018). IEEE Access. PP. 1-1. 10.1109/ACCESS.2018.2841321.
(c) - Detecting the dynamics of urban structure through spatial network analysis by Chen Zhong and Stefan Müller Arisona and Xianfeng Huang and Michael Batty and Gerhard Schmitt, International Journal of Geographical Information Science Volume 28, 2014 - Issue 11. https://doi.org/10.1080/13658816.2014.914521
(d) - The Size Distribution of 'Cities' Delineated with a Network Theory-based Method and Smartphone GPS Data by Shota Fujishima, Naoya Fujiwara, Yuki Akiyama, Ryosuke Shibasaki, and Hodaka Kaneda, Center for Spatial Information Science, Discussion Paper http://www.csis.u-tokyo.ac.jp/wp-content/uploads/2018/02/150.pdf

This is not a comprehensive list of the topic(I just searched in a web). Please add a short review in Introduction and give sufficient credits to such previous works. I also think this paper contains original results to be published, because of the new method of community detection with the application to another dataset.

Addressed: Many many thanks to the referee and apologies for this omission; we have now gone back and done a much more extensive literature search. We have divided the "Related work" section into two subsections: one dealing specifically with related literature on region demarcation and the second a wider discussion on related work using network techniques especially community detection techniques for the analysis of spatial urban networks. We discuss these works in the newly created section 2.2 (approximately paragraph).

This exercise has also significantly improved our own education.

(3) Concerning the community detection method, I think the reliability is not checked enough to use it for the purposes addressed in this paper. The authors aimed to identify spatial areas, instead of CBSA(core based statistical areas) or MSA(metropolitan areas), for examples. Such areas are the basis of many statistics in US, and should be carefully considered to guarantee the reliability of the detection method. The community detection methods, however, have many problems in general. This is mainly because the likelihood function is multimodal and there are a number of near-optimal partitions, as discussed in literature.
(a) - B.H. Good, Y.-A. de Montjoye and A. Clauset, "The performance of modularity maximization in practical contexts." Physical Review E 81, 046106 (2010).
(b) -Peel, Leto & B. Larremore, Daniel & Clauset, Aaron. (2016). The ground truth about metadata and community detection in networks. Science Advances. 3. 10.1126/sciadv.1602548. This would be problematic to obtain reliable results.

I suspect that the author's optimization method focuses only a single partition that would be optimal, and can not deal with the problem property.

Recently, network scientists have examined the ensembles of partitions, not just focusing on the optimal one. For a example, the number of clusters are determined by the model selection or model evidence in Bayesian inference framework. - A review, Tiago P. Peixoto, "Bayesian stochastic blockmodeling", arXiv: 1705.10225. It would be better if the authors carefully consider this issue and develop their

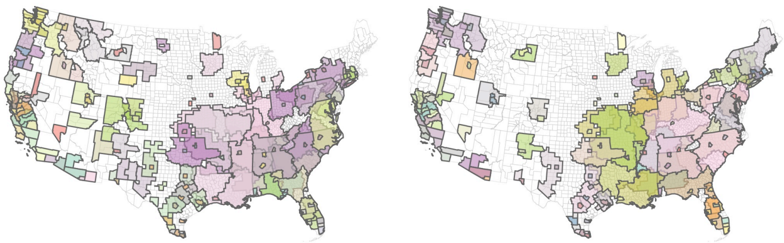

FIGURE 0.1. CCME-SL algorithm run under differing initializations using thresholding of self-loops (*left*: $W_{uu} > 10,000$,*right*: $W_{uu} > 50,000$ and α. The resultant communities do not differ much from those initialized with the threshold 20,000

method. At least, the authors should address the issue in Discussion and argue on the potential limitations of their methods.

Addressed:

We appreciate and acknowledge the reviewer's concerns for ensembles of partitions as most clustering algorithms. We have addressed this point in the discussion section in section 7.3 (paragraph 505).

The proposed method is not strongly affected by the reviewer's concerns in two ways. Firstly, the initialization heuristic is fixed. Secondly, even under varying settings for initialization, the resultant clusters do not differ much (figure B in supplement shows resultant clusters when initialized from the 10,000 and 50,000 thresholds). This is partly due to the filtering-of-overlaps portion of the algorithm, which removes clusters that have too many overlapping nodes: counties that are near each other lead to similar clusters, which are in turn filtered out. Results are shown here as well as in the supplement.

Because our method is initialized using a fixed heuristic of starting from counties that are are population centers, our proposed method, is able to reach a fixed, convergent result. The choice of initialization is not randomized because they are based in interpretable processes of commuting behavior: we start from nodes of highest commuting volumes, and start the iterative search procedure from these nodes in an outward manner starting from the *core* and then scanning for the *peripheral* repeatedly. Such a fixed initialization scheme is intuitive with the workings of the iterative search procedure as well as in the context of this application.

Though most iterative methods for community detection understandeably initialize randomly, specific to this application we choose fixed points representing populous counties. As such, we are able to procure the same results every time.

Therefore, though the (analog to) likelihood in our approach may be likely multimodal, befcause our initialization heuristic is fixed in an interpretable way, we derive the same communities every time.

In other applications of the CCME or CCME-SL algorithm where the initialization is not fixed, and where initialization from random seedings may lead to different results, deeper consideration of the issues of *sets of optimal partitions* will need to be addressed.

(4) The proposed method is not clear, because

(i) Two different parts of the method are mixed. The theoretical description of the statistical model and its numerical implementation should be described separately.

(ii) The relation to the previous models is unclear.

Related to the second point, the proposed method seems to be non-standard in network science. This is okay, but I would like to ask about the ***resolution limit***, that is already studied in the cases of standard methods. This resolution limit is significantly related to the issue of the detection of 'Monads', obtained by self-loop consideration. Is the limit is small enough to detect a single node as a community? Moreover, the authors are responsible to allocate their method in literature to join the scientific discussions. The current manuscript is not enough for readers to understand the relationships to previous methods, related to the point (i). For example, could you formulate the model in the Bayesian framework and argue the differences from degree-corrected SBM?

Addressed: Regarding the first point, we have separated the sections in section 4.2.1 to better demarcate the theoretical component of the methodology and the iterative algorithm. Section 5 only describes the numerical procedure. Regarding the second point, note that the both the definition of "monad" and the corresponding extraction of monads is **different** from the extraction of single node communities as the proposed methodology compares the "self-loopiness" of each vertex against a baseline level of self-loopiness and uses this to extract subroutines; thus monads are different from possibly single node communties that could be extracted by the community detection subroutine. Your other points are extremely pertinent and thank you for drawing our attention to this omission. We have included a full discussion on the resolution limit in the discussion section in the limitations of the method and have also outlined work in progress that we hope will mitigate at least some of these issues in the spatial context case including the modification of the resolution parameter work of Reichardt and Bornholdt. Finally regarding your last comment about describing the relationship of this methodology to the main body of community detection, you are absolutely right; we have now added an entire subsection (Section 7.4) describing this connection.

(5) The details of the proposed model seems to be unnatural to model the commuting data. In the null model, adjacency matrix A and weight matrix W is separately used. (After assigning edges, the weight is allocated only to existing edges.) But, the commuting data is represented only by W, and A is just its binarization by a threshold of 100 commuters. Why not consider a model of W, without A? What is the aim to introduce A? Please explain the logic behind the modelling. In addition, the threshold seems to be ad-hoc.

Addressed: The weight matrix and adjacency matrix represent inherently different, though correlated modes of relation. For example in a social network one can imagine two individuals having similar degree but very different modes (for example rates of interaction) with the respective friends of these individuals. In the context of our commuting data, Mesa County, CO and L.A. County, CA, have similar degree but very different strengths of interaction. Thus part of the aim of this paper was to develop a baseline null model that would preserve both degrees and strengths as well as a baseline level of self-loopiness and then compare an empirically observed network against this null model to extract regions of significantly higher connectivity after accounting for this baseline connectivity. We have now included a paragraph describing this in the writeup in Section 4.2.2 (paragraph 170.)

(6) Authors argued the novelty of introduction of self-loops to community detection methods. But, I think, this is not novel. The previous studies have also considered it, and in most cases, there is no limitation on self-loops, except that the methods are based on tree-like approximations in sparse graphs. It just depends on datasets in practice. I agree that the self-loops will be important in the commuting data studied in this paper.

But, the self-loops will not so important in higher-resolution mobility data, such as 500mx500m grid.

Today, because of GPS and other technologies, we are getting such nice datasets, and in the cases, the commuters within the same county are recorded as those between different locations and self-loops will be rare.

Please rewrite your arguments on the novelty considering these points.

Addressed:

Though other methods often consider the role of self loops, our method is among the first to have integrated it in a iterative testing framework. Previous tree-based methods do mention self-loops, but few that focus explicitly on strongly self looping networks that account for over 50 % of the weights.

One such tree-based method is "Multi-resolution community detection based on generalized self-loop rescaling strategy" by Xiang et al. 2015, which uses a modularity measure that is rescaled by the size of the self loop. The paper suggested by the review written Peixoto (2017) allow for self loops in Bayesian stochastic blockmodel formulation. Another paper that accounts for self loops is Cafieri et al (2016) in the context of modularity maximization.

Existing methods that simply *allow* for self loops, or presume that self-loops are somehow *similar* in characteristic to the other edges, sometimes do not properly account, or break down, when the self loops are too large, as in the case of the existing CCME algorithm introduced by Palowitch et al. To our knowledge, CCME-SL is the only method that is specifically tailored to a predominantly self looping network.

Empirically, if the self-loop has for example *much* larger variance amongst self loops, the method for clustering would be expected to break down under the existing approaches. In the case of a generative blockmodel, if the cross-edges adhere to a Poisson distribution for cross-edges but a power law distribution for self loops, we can expect the clustering method to break down if it presumes that all cross edges and self-looping edges arise from the same generative distributions.

Just as Karrer and Newman (2010) have shown that high-degree nodes tend to distort clustering in a stochastic blockmodel that would necessitate degree correction, running 'ordinary' community detection in a significantly self-looping network would produce distorted resultant clusters. CCME-SL is able to account for the power-law nature of a graph whose heavy tails originate from self loops. This type of network is perhaps most immediately realized in the commuting network. We have added a review of the literature in section 4.2 (paragraph 168) that discuss the existing literature on self-loops.

The reviewer is right to bring up the continuously evolving quality of network data in terms of resolution. It is true that resolutions are becoming finer, however much of the federal and state policies still rely on agglomerations of political units such as counties. According to a report by Brookings Institute, "Not simply statistical arcana, OMB-defined metropolitan and micropolitan areas have real-worldimplications for public- and private-sector research, for federal programs—think Medicare, Section 8 housing assistance—and for how big-city and small-town residents view the places thatthey live." See https://www.psc.isr.umich.edu/dis/census/freybrookings.pdf

(7) I would like to see the result of the community detection in entire US, and visually compare it with the other partitions of MBA, megaregions, and other partitions of standard methods (if possible. e.g. degree-corrected SBM and modularity). Then, the results of the paper would be more effectively presented, and facilitate further scientific discussions.

It is possible plot the overlapping communities using pi charts. https://graph-tool.skewed.de/static/doc/_images/lesmis-sbm-marginals.svg

Addressed:

We have added a figure (figure 6) and a corresponding section (section 6.2: Comparison with other methods paragraphs 456-462) describing the clusters ascertained from other methods. We used the degree-corrected SBM as well as the modularity algorithm, both accounting for self loops. We have also moved the figure showing existing delineations from MSA and megaregions from figure 2 to figure 6 to show a comprehensive 'comparison figure'. We have opted to not include the overlap charts because CCME-SL produces too many communities for this tool to be utilized effectively.

Other changes. We now describe the other changes in the revision:

(i) We have gone through the entire paper and tried to improve the exposition.
(ii) Owing to the comments of the other referee and the associate editor, we have now included a more extensive discussion of natural possible future extensions of the work in this paper (Section 3.2).
