# Supplementary material for: Demarcating geographic regions using community detection in commuting networks with significant self-loops
Source: PLoS One. 2020 Apr 29;15(4):e0230941. doi: 10.1371/journal.pone.0230941 (PMC7190107; doi:10.1371/journal.pone.0230941)
Supplement: Attachment — Submitted filename: PLOS_ReSub2_RevComments2.docx [file pone.0230941.s005.docx]

**Reviewer #1:** The revised manuscript has addressed the issues I pointed out, and the authors have responded them carefully. I still have some different opinions on the issues, but I also think that such differences are not critical and should be judged by readers.

However, there is one exception related to the point (3). The authors argued that the output of the method is stable if the initial condition is fixed, and the outputs are robust against the change of the initial conditions. This is necessary requirement, but, it is not enough.

To clarify the addressed issue, I'd like to focus on a single aspect of the output of the method. This is the number of the detected community by their method. This number is very important and should be determined on a reasonable basis. For example, in DC-SBM, the number can be determined by model selection or by cross validation (in this paper the number is pre-determined by authors in sec. 6. This should be updated, too). On the other hand, in the proposed method, the number of detected communities is determined by just one sample. This would be one of near optimal states of the assumed model, but there would a lot of another optimal states with different numbers of communities. Thus, there is no reasonable validation whether the detected number is good or not.

I think this is a difficult problem, and my question did not mean that this issue should be solved perfectly. I also think that the proposed method is good enough to present a new visualization tool of geographic regions. But, this point can be problematic if this paper is still aimed to propose a new identification method for grouping counties for further statistics, and such limitations should be noted.

We agree with the concerns raised by the referee. The number of communities is non-trivial issue. We have reworded the text to emphasize that the method developed is for exploratory purposes of clustering regions that provide an exploratory avenue that would give rise to further work towards the goal of officially designating statistical areas. We have added this in the paragraph in section 6.3 on page 24. We have also added the text describing the importance of these issues in future exploration of this methodology. We thank the reviewer for pointing this out.

**Reviewer #2:** I thank the authors for addressing most of my concerns and I think that the manuscript is much improved. I have a few final comments.

1. I did not mean that monad should be changed for 'self-explanatory single-node community' which I agree is a mouthful. I was suggesting 'single-node community' and I meant that this term was self-explanatory. I still have the sense that introducing so much new terminology as the authors do makes is less intelligible to their peers. However, this is just a suggestion and I leave it now to their best judgement after having explained myself.

We thank the reviewer for pointing this out, but we left the term “monad” to contrast with “dyad”.

2. While the manuscript is much improved in the explanation of the terms, there are still a couple of things that are confusing. In the abstract the term 'non-trivial' to refer to self-loops is really confusing. What is a trivial self-loop? We have to wait until page 12 to find out, which is very bad practice for an abstract. Idem for the term propensity. It is defined in page 6 but discussed much before then. **This is also not a standard term, and I would suggest removing it entirely and explaining instead that you mean the proportion of the inter-county commuting flow compared to the total outward flow.**

We agree. We have removed *“non-trivial self-loop”* from the abstract and cleaned up the abstract and the text. We used propensity simply to mean “tendency”, but we have clarified the definition. In page 6 we have more clearly defined the term *non-trivial self-loop* and we made sure that previous parts of the manuscript do not refer to it. We have also defined *Strong Self-looping Network* in section 2 (page 6).

3. The authors discuss the other community detection methods in terms of a comparison with a null model. They imply that this is done within the SBM framework, but this is not the case. In this framework we decide to model the data with a particular type of model, and then we fit the model parameters. In page 5 they talc about the 'structured SBM' and I don't know what structured means here.

We thank the reviewer for pointing this out. We have edited the manuscript in page 5 to remove the term ‘structured SBM’ as well as in other sections to reflect the changes and to clarify the wording.

4. I would swap the sections 4.1 and 4.2 because the notation comes before it is needed, and more discussion follows.

We thank the reviewer for pointing this out and we have switched the sections.

5. A minor point, but I see no technical reason why the degree of node u needs to exclude self-loops (the degree is fact is almost not used). To make this more similar to the strength which does include node u, it is convenient to define A_uu = 2 as a convention, and in this case the normal degree formulas work and the degree of each node (with self-loops) simply increases by 2.

We thank the reviewer for making a good point. The technical reason for why node u needs exclude self-loops is to satisfy the assumptions of the weighted configuration model such that the estimated degrees (sum of estimated weights) all sum to the actual degrees. We have now added a line in page 7 to describe that

“Though conventionally the self-loop edge is defined as $A_{uu}=2$, we define it to be 1 as this convention makes the algebra simple when defining the null model.”

6. While some changes have been made and the authors acknowledge that the SBM methodology accommodates for self-edges, the text still says that most other methods and in particular modularity in general does not account for self-loops. This is not the case, and in fact the Newman-Girvan null model implicitly allows self-loops.

We thank the reviewer for pointing this out and we did not mean to imply other methods do not account for self-loops, rather our method was motivated from the outset to deal with self-looping networks and that produces meaningful results when the self-loops are extremely large, as is in the case of spatial commuting networks.

We have clarified several sections of the manuscript that may have confusingly stated that CCME-SL is the only model that accounts for self-loops. We acknowledge that the Louvain and DC-SBM methods also implicitly account for self-loops. Notably in the beginning of the *Discussion* (section 6) in page 22, we

We highlight how and why our findings are different from typical delineations and reconcile such findings with the novelty of the introduced method in its incorporation of self-loops in a null model within a weighted network.

In the first paragraph of the *Comparison with Other Methods (*section 5.2) on page 20, we have added:

A number of other techniques implicitly allow for self loops. CCME-SL was largely motivated for settings where self-loops account for a significant proportion of the weight emanating from a vertex.

We have also implemented the Newman -Girvan method, which implicitly accounts for self-loops, and the results were less optimal from from a domain perspective. We have attached the results and a brief discussion here:

Results from the Newman-Girvan algorithm implemented from the \textit{igraph} package are shown in the following figure. Unlike DC-SBM or Louvain, the sizes of the clusters are very large in range. Most of the clusters are singletons but there are 4 or 5 massive clusters that span nearly all of the Northeast, Texas, Mississippi, Wisconsin, and the Midwest.

7. In page 10 there are two instance of \hat{\rho}_u that seem to be referring to two different quantities, an empirical and a simulated one.

We have corrected the error in page 10.

***8.*** Figure 6 should be moved before the references. This figure really adds to the manuscripts and in particular it is easy to see that the other methods find a very different structure (for example the 'background noise' that their method disregards). I would point out in the text in section 6.2 that the methods give non-overlapping communities (i.e. partitions as opposed to covers) (althought the SBM can be used to get covers, but this is probably much more work and not necessary).

We had originally intended for the figure to be before the appendices but it was moved due to an artifact of it being floating object in Latex. We have moved it to the section that describes the different methods.

***9.*** I did not understand the point of section 7.4, which is completely out of place with the discussion before and after (notably with the definition of modularity).

We thank the reviewer for pointing out that this paragraph does not flow well with surrounding text in the manuscript. We have rewritten the section and moved part of it to sections 1 and 2. We also added discussion of competing methods in the section.

***10***. Final comment, the text still needs more a good proofread. There a new errors (still missing or extra words), and in the new section 2.2 the authors cannot use '[2]' as a subject, but they can write 'In reference '[2]', the authors do X and Y'. The numbers cannot be used as a subject in the sentence.

We have carefully checked all the references and have corrected the bad notation when referring to references. In particular in section 1.3 (previously section 2.1) in page 4, which discusses the prior work on spatial networks. We have thoroughly proofread the paper.
